# Ordinal Pattern Based Entropies and the Kolmogorov–Sinai Entropy: An Update

**DOI:** 10.3390/e22010063

**Published:** 2020-01-02

**Authors:** Tim Gutjahr, Karsten Keller

**Affiliations:** Institute of Mathematics, University of Lübeck, D-23562 Lübeck, Germany; keller@math.uni-luebeck.de

**Keywords:** ordinal patterns, Kolmogorov–Sinai entropy, permutation entropy, conditional entropy

## Abstract

Different authors have shown strong relationships between ordinal pattern based entropies and the Kolmogorov–Sinai entropy, including equality of the latter one and the permutation entropy, the whole picture is however far from being complete. This paper is updating the picture by summarizing some results and discussing some mainly combinatorial aspects behind the dependence of Kolmogorov–Sinai entropy from ordinal pattern distributions on a theoretical level. The paper is more than a review paper. A new statement concerning the conditional permutation entropy will be given as well as a new proof for the fact that the permutation entropy is an upper bound for the Kolmogorov–Sinai entropy. As a main result, general conditions for the permutation entropy being a lower bound for the Kolmogorov–Sinai entropy will be stated. Additionally, a previously introduced method to analyze the relationship between permutation and Kolmogorov–Sinai entropies as well as its limitations will be investigated.

## 1. Introduction

The Kolmogorov–Sinai entropy is a central measure for quantifying the complexity of a measure-preserving dynamical system. Although it is easy from the conceptional viewpoint, its determination and its estimation from given data can be challenging. Since Bandt, Keller, and Pompe showed the coincidence of Kolmogorov–Sinai entropy and permutation entropy for interval maps (see [1]), there have been different attempts to approach the Kolmogorov–Sinai entropy by ordinal pattern based entropies (see e.g., [2,3,4,5,6] and references therein), leading to a nice subject of study. In this paper, we want to discuss the relationship of the Kolmogorov–Sinai entropy to the latter kind of entropies. We respond to the state of the art and give some generalizations and new results, mainly emphasizing combinatorial aspects.

For this, let (Ω,A,μ,T) be a *measure-preserving dynamical system*, which we think to be *fixed in the whole paper*. Here, (Ω,A,μ) is a probability space equipped with a A-A- measurable map T:Ω→Ω satisfying $\mu (T^{-1}\left(A\right))=\mu \left(A\right)$ for all A∈A. Certain properties of the system will be specified at the places where they are of interest. It is suggested for the following to interpret Ω as the set of states of a system, μ as their distribution, and *T* as a description of the dynamics underlying the system and saying that the system is in state T(ω) at time t+1 if it is in state ω∈Ω at time *t*.

In the following, we give the definitions of the central entropies considered in this paper.

### 1.1. The Kolmogorov–Sinai Entropy

The base of quantifying dynamical complexity is to consider the development of partitions and their entropies under the given dynamics. Recall that the coarsest partitions refining given partitions $P_{1},P_{2},\ldots ,P_{k}$ and P,Q of Ω are defined by

$$\overset{k}{\underset{s=1}{⋁}}P_{s}:=\left\{\overset{k}{\underset{s=1}{⋂}}P_{s}\neq \emptyset |P_{s}\in P_{s}\;\mathrm{for}\;s=1,2,\ldots ,k\right\}$$

and

P∨Q:={P∩Q≠∅∣P∈P,Q∈Q},

respectively. The entropy of a finite or countably infinite partition Q⊂A of Ω is given by

$$H\left(Q\right):=-\sum_{Q\in Q}\mu \left(Q\right)\log\mu \left(Q\right).$$

For a finite or countably infinite partition $P:={\left\{P_{i}\right\}}_{i\in I}\subset A$ of Ω and some k∈N, consider the partition

$$P^{\left(k\right)}:=\overset{k-1}{\underset{t=0}{⋁}}T^{-t}\left(P\right)=\left\{P\left(i\right)\neq \emptyset |i\in I^{k}\right\},$$

where

$$P\left(i\right):=\overset{k-1}{\underset{t=0}{⋂}}T^{-t}\left(P_{i_{t}}\right)$$

for each multiindex $i=\left(i_{0},i_{1},\ldots ,i_{k-1}\right)\in I^{k}$. The *entropy rate* of *T* with regard to a finite or countably infinite partition P⊂A with H(P)<∞ is defined by

$$h\left(P\right):=\lim_{n\to \infty }\frac{1}{n}H\left(P^{\left(n\right)}\right).$$

The *Kolmogorov–Sinai entropy* is then defined as

$$\mathrm{KS}:=\underset{P}{\sup}h\left(P\right),$$

where the supremum is taken over all finite or over all countably finite partitions P⊂A with H(P)<∞.

### 1.2. Ordinal Pattern Based Entropy Measures

As the determination and estimation of the Kolmogorov–Sinai entropy based on the given definition are often not easy, there are many different alternative approaches to it, among them the permutation entropy approach by Bandt and Pompe [7]. The latter is built up on the concept of ordinal patterns, which we describe in a general manner now.

For this, let $X=\left(X_{1},X_{2},\ldots ,X_{d}\right):\Omega \to R^{d}$ be a random vector for d∈N. Here, each of the random variables $X_{i}$ can be interpreted as an observable measuring some quantity in the following sense: If the system is in state ω at time 0, then the arschvalue of the quantity mesured at time *t* provides $X_{i}\left(T^{t}\left(\omega \right)\right)$. This general approach includes the one-dimensional case that states and measurements coincide, and this is that Ω⊆R and X=id is the identical map on Ω. This case, originally considered in [7] and subsequent papers, is discussed in Section 3. We refer to it as the *simple one-dimensional case*.

**Figure 1:**
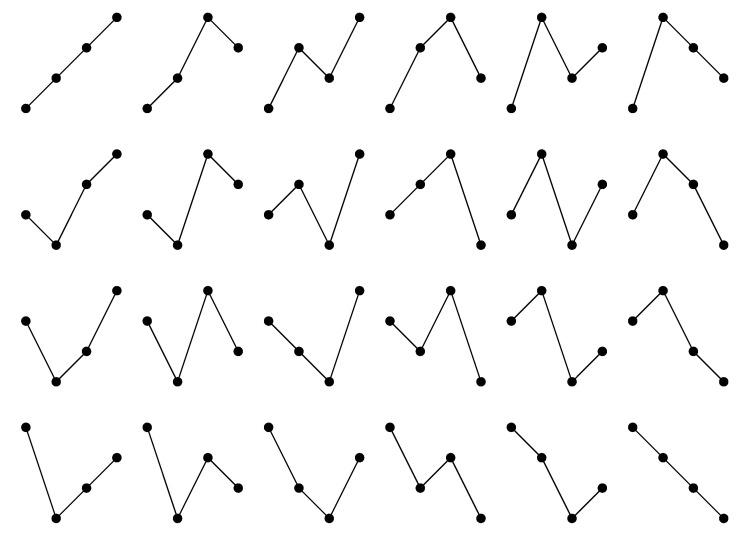
Abstract visualization of all 24 possible ordinal patterns of length 4.

Let

$$\Pi_{n}:=\left\{\left(r_{0},r_{1},\ldots r_{n-1}\right)\in {\left\{0,1,\ldots n-1\right\}}^{n}|r_{i}\neq r_{j}\;\mathrm{for}\;i\neq j\right\}$$

be the set of all permutations of length *n*. We say that a vector $\left(x_{0},x_{1},\ldots ,x_{n-1}\right)\in R^{n}$ has ordinal pattern $\pi =\left(r_{0},r_{1},\ldots r_{n-1}\right)\in \Pi_{n}$ if

$$x_{r_{i-1}}<x_{r_{i}}\;\mathrm{or}\;x_{r_{i-1}}=x_{r_{i}}\;\mathrm{and}\;r_{i}<r_{i-1}$$

holds true for all i∈{1,2…,n−1}. The n! possible ordinal patterns (compare Figure 1) provide a classification of the vectors. We denote the set of points with ordinal pattern $\pi_{1},\pi_{2},\ldots ,\pi_{d}\in \Pi_{n}$ with regard to $X_{1},X_{2},\ldots ,X_{d}$, respectively, by

$$\begin{array}{c} \;P_{\pi_{1},\pi_{2},\ldots ,\pi_{d}}^{X}\\ =\left\{\omega \in \Omega |\left(X_{i}\left(\omega \right),X_{i}\left(T\left(\omega \right)\right),\ldots ,X_{i}\left(T^{n-1}\left(\omega \right)\right)\right)\;\mathrm{has}\;\mathrm{ordinal}\;\mathrm{pattern}\;\pi_{i}\;\mathrm{for}\;i=1,2,\ldots ,d\right\} \end{array}$$

and by

$$O\;P^{X}\left(n\right):=\left\{P_{\pi_{1},\pi_{2},\ldots ,\pi_{d}}^{X}:\neq \emptyset |\pi_{1},\pi_{2},\ldots ,\pi_{d}\in \Pi_{n}\right\}$$

the partition of Ω into those sets. 

We are especially interested in three ordinal pattern based entropy measures. These are the *lower* and *upper permutation entropies* defined as

$${\underline{\mathrm{PE}}}^{X}=\underset{n\to \infty }{lim inf}\frac{1}{n}H\left(O\;P^{X}\left(n\right)\right)$$

and

$${\bar{\mathrm{PE}}}^{X}=\underset{n\to \infty }{lim sup}\frac{1}{n}H\left(O\;P^{X}\left(n\right)\right),$$

respectively, and the *conditional entropy of ordinal patterns* defined by

$${\mathrm{CE}}^{X}=\underset{n\to \infty }{lim inf}\;\left[H\left({\left(O\;P^{X}\left(n\right)\right)}^{\left(2\right)}\right)-H\left(O\;P^{X}\left(n\right)\right)\right].$$

We speak of the *permutation entropy* if the upper and lower permutation entropies coincide.

### 1.3. Outline of This Paper

In Section 2, we will focus on the relationship between permutation and Kolmogorov–Sinai entropies in the general setting. With Theorems 1 and 3, we will restate two known statements. A new proof of Theorem 1 will be given in Appendix A.2. Theorem 3 is stated for completeness. Theorem 2 establishes a new relationship between the conditional permutation entropy and the Kolmogorov–Sinai entropy.

In Section 3, the relationship between permutation and Kolmogorov–Sinai entropies in the one-dimensional case is investigated. Conditions are introduced, under which the permutation entropy is equal to the Kolmogorov–Sinai entropy. The given conditions allow for a generalization of previous results. We will explain why (countably) piecewise monotone functions satisfy these conditions and consider two examples.

In Section 4, we will investigate a method to analyze the relationship between permutation and Kolmogorov–Sinai entropies that was first introduced in [5]. We will use this method to relate two different kinds of conditional permutation entropies in the general setting. Theorem 5 shows that this method cannot be used directly to prove equality between permutation and Kolmogorov–Sinai entropies.

The results of the paper are summarized in Section 5. The proofs for all new results can be found in the Appendix A.

## 2. Relating Entropies

### 2.1. Partitions via Ordinal Patterns

Given some d,n∈N and some random vector $X=(X_{1},X_{2},\ldots ,X_{d})$, the partition described above can be defined in an alternative way, which is a bit more abstract but better fitting for the approach used in the proof of Theorem 4:

We can determine to which set $P_{\pi }\in O\;P^{X_{i}}\left(n\right)$ a point ω∈Ω belongs to if we know whether $X_{i}\left(T^{s}\left(\omega \right)\right)<X_{i}\left(T^{t}\left(\omega \right)\right)$ holds true for all s,t∈{0,1,…,n−1} with s<t. Therefore, we can write

$$O\;P^{X_{i}}\left(n\right)=\overset{n-1}{\underset{s=0}{⋁}}\overset{n-1}{\underset{t=s+1}{⋁}}\left\{\left\{\omega \in \Omega |X_{i}\left(T^{s}\left(\omega \right)\right)<X_{i}\left(T^{t}\left(\omega \right)\right)\right\},\left\{\omega \in \Omega |X_{i}\left(T^{s}\left(\omega \right)\right)\geq X_{i}\left(T^{t}\left(\omega \right)\right)\right\}\right\}.$$

Throughout this paper, we will use the set

$$R:=\{\left(x,y\right)\in R^{2}|x<y\}$$

to describe the order relation between two points. This notation allows us to write

$$O\;P^{X}\left(n\right)=\overset{d}{\underset{i=1}{⋁}}\overset{n-1}{\underset{s=0}{⋁}}\overset{n-1}{\underset{t=s+1}{⋁}}{\left(T^{s},T^{t}\right)}^{-1}\left({\left(X_{i}\times X_{i}\right)}^{-1}\left(\{R,R^{2}\backslash R\}\right)\right).$$

### 2.2. Ordinal Characterization of the Kolmogorov–Sinai Entropy

To be able to reconstruct all information of the given system via quantities based on the random vector $X=\left(X_{1},X_{2},\ldots ,X_{d}\right)\to R^{d}$, we need to assume that the latter itself does not reduce information. From the mathematical viewpoint, this means that the σ-algebra generated by X is equivalent to the originally given σ-algebra A, i.e., that

(1) $$\sigma \left(\left\{X_{i}\circ T^{t}|t\in N_{0},i\in \left\{1,2,\ldots ,d\right\}\right\}\right)\overset{\mu }{=}A$$

holds true, which is roughly speaking that orbits are separated by the given random vector. For definitions and some more details concerning σ-algebras and partitions, see Appendix A.1.

The following statement saying that, under (1), ordinal patterns entailing the complete information of the given system have been shown in [3] in a s slightly weaker form than given here.

**Theorem** **1.** *Let $X:\Omega \to R^{d}$ be a random vector satisfying (1). Then,*

(2) $${\underline{\mathrm{PE}}}^{X}\geq \lim_{k\to \infty }h\left(O\;P^{X}\left(k\right)\right)=\mathrm{KS}$$

*holds true.*

Note that the inequality in (2) is a relatively simple fact: Since the partition $O\;P^{X}\left(n\right)$ is finer than the partition ${\left(O\;P^{X}\left(k\right)\right)}^{\left(n-k\right)}$ for all n≥k, we have

$$H\left(O\;P^{X}\left(n\right)\right)\geq H\left({\left(O\;P^{X}\left(k\right)\right)}^{\left(n-k\right)}\right).$$

Dividing both sides by *n* and taking *n* and subsequently *k* to infinity proves this inequality.

Proofs of the inequality ${\underline{\mathrm{PE}}}^{X}\geq \mathrm{KS}$ are also implicitly given in [1,8]. One-dimensional systems with direct observation as considered there are discussed in Section 3 in detail.

We will give a proof of the equality in (2) in Appendix A.2 being alternative to that in [3].

### 2.3. Conditional Entropies

In the case that (1) holds and that KS and ${\underline{\mathrm{PE}}}^{X}$ coincide, in Appendix A.3, we will prove different representations of the Kolmogorov–Sinai entropy by ordinal pattern based conditional entropies as they are given in the following theorem.

**Theorem** **2.** *Let $X:\Omega \to R^{d}$ be a random vector satisfying (1). If $\mathrm{KS}\geq {\bar{\mathrm{PE}}}^{X}$ is true, then*

$$\begin{array}{cc} \mathrm{KS} & =\underset{n\to \infty }{lim inf}H\left(O\;P^{X}\left(n\right)|T^{-1}\left({\left(O\;P^{X}\left(n\right)\right)}^{\left(k\right)}\right)\right)\\ \; & =\underset{n\to \infty }{lim inf}H\left({\left(O\;P^{X}\left(n+1\right)\right)}^{\left(k\right)}|{\left(O\;P^{X}\left(n\right)\right)}^{\left(k\right)}\right)\\ \; & ={\underline{\mathrm{PE}}}^{X}={\bar{\mathrm{PE}}}^{X} \end{array}$$

*holds true for all k∈N, in particular, in the case k=1, one has $\mathrm{KS}={\mathrm{CE}}^{X}={\underline{\mathrm{PE}}}^{X}={\bar{\mathrm{PE}}}^{X}$.*

### 2.4. Amigó’S Approach

Amigó et al. [2,8] describe an alternative ordinal way to the Kolmogorov–Sinai entropy, which is based on a refining sequence of finite partitions. We present it in a slightly more general manner as originally given and in the language of finite-valued random variables. Note that the basic result behind Amigo’s approach in [2,8] is that the Kolmogorov–Sinai entropy of a finite alphabet source and its permutation entropy given some order on the alphabet coincide (see also [9] for an alternative algebraic proof of the statement).

**Theorem** **3.** *Given a sequence ${\left(X_{k}\right)}_{k\in N}$ of R-valued random variables satisfying*

*(i)*  *$\#(X_{k}\left(\Omega \right))<\infty$ for all k∈N,*
*(ii)*  *$\sigma \left(X_{k}\right)\subseteq \sigma \left(X_{k+1}\right)$ for all k∈N,*
*(iii)*  *$\sigma \left(\left\{X_{k}|k\in N\right\}\right)\overset{\mu }{=}A$,*

*then it holds*

$$\lim_{k\to \infty }{\underline{\mathrm{PE}}}^{X_{k}}=\mathrm{KS}.$$

## 3. The Simple One-Dimensional Case

In the following, we consider the case that Ω is a subset of R with A coinciding with the Borel σ-algebra B on Ω, and with X=id being the identical map on Ω. The X is superfluous here, which is why we leave out each superscript X. For example, we write OP(n) instead of $O\;P^{id}\left(n\right)$.

### 3.1. (Countably) Piecewise Monotone Maps

We discuss some generalization of the results of Bandt, Keller, and Pompe that Kolmogorov–Sinai entropy and permutation entropy coincide for interval maps (see [1]) on the basis of a statement given in the paper [10]. The discussion sheds some light on structural aspects of the proofs given in that paper with some potential for further generalizations.

**Definition** **1.** *Let* Ω *be a subset of R and B be the Borel σ-algebra on* Ω *and μ be a probability measure on (Ω,B). Then, we call a partition $M={\left\{M_{i}\right\}}_{i\in I}$ of* Ω *ordered (with regard to μ), if M⊂B and*

(3) $$\mu^{2}\left((M_{i_{1}}\times M_{i_{2}})\cap R\right)\in \left\{0,\mu^{2}\left(M_{i_{1}}\times M_{i_{2}}\right)\right\}$$

*holds true for all $i_{1},i_{2}\in I$ with $i_{1}\neq i_{2}$. Here, $\mu^{2}$ denotes the product measure of μ with itself.*

*We call a map T:Ω→Ω (countably) piecewise monotone (with regard to μ) if there exists a finite (or countably infinite) ordered partition $M={\left\{M_{i}\right\}}_{i\in I}$ of* Ω *with H(M)<∞ such that*

(4) $$\mu^{2}\left(\left(M_{i}\times M_{i}\right)\cap R\cap {\left(T\times T\right)}^{-1}\left(R\right)\right)\in \left\{0,\mu^{2}\left((M_{i}\times M_{i})\cap R\right)\right\}$$

*holds true for all i∈I.*

Given a probability space (Ω,A,μ), for two families of sets P,Q⊆A, we write

P≺Q

if, for all Q∈Q, there exists a P∈P with μ(Q\P)=0. If $P={\left\{P_{i}\right\}}_{i\in I}$ and $Q={\left\{Q_{j}\right\}}_{j\in J}$ are partitions of Ω in A, P≺Q is equivalent to the fact that for every i∈I there exists a set $J_{i}\subseteq J$ such that $P_{i}$ and ${⋃}_{j\in J_{i}}Q_{j}$ are equal up to some set with measure 0.

Moreover, given a partition $M={\left\{M_{i}\right\}}_{i\in I}$ of a set Ω, let

$$M^{\left(m\right)}⊗M^{\left(m\right)}:=\left\{M_{i_{1}}\times M_{i_{2}}|M_{i_{1}},M_{i_{2}}\in M^{\left(m\right)}\right\}.$$

In Appendix A.4, we will show the following statement:

**Theorem** **4.** *Let* Ω *be a subset of R and A=B be the Borel σ-algebra on* Ω*, and assume that the following conditions are satisfied:*

**Condition 1:** *There exists a finite or countably infinite ordered partition $M={\left\{M_{i}\right\}}_{i\in I}\subset B$ with H(M)<∞ and some m∈N with*

(5) $$M^{\left(m\right)}⊗M^{\left(m\right)}\vee \left\{R,\Omega^{2}\backslash R\right\}\;≺\;M^{\left(m\right)}⊗M^{\left(m\right)}\vee \overset{m}{\underset{u=1}{⋁}}{\left(T\times T\right)}^{-u}\left(\{R,\Omega^{2}\backslash R\}\right).$$

**Condition 2:** *For all ϵ>0, there exists a finite or countably infinite ordered partition Q with H(Q)<∞ and*

(6) $$\sum_{Q\in Q}\underset{n\to \infty }{lim sup}\frac{1}{n}\sum_{l=1}^{n}\mu \left(Q\cap T^{-l}\left(Q\right)\right)<\epsilon .$$

*Then,*

$$\bar{\mathrm{PE}}\leq \mathrm{KS}$$

*holds true.*

Theorem 4 extracts the two central arguments in proving the main statement of [10] in the form of Conditions 1 and 2. This statement is given in a slightly stronger form in Corollary 1. In the proof of [10], the *m* in Condition 1 is equal to 1. We will discuss in Section 3.2 a situation where Condition 1 with m=2 is of interest.

**Corollary** **1.** *Let* Ω *be a compact subset of R and A=B be the Borel σ-algebra on* Ω*. If T is (countably) piecewise monotone, then*

$$\bar{\mathrm{PE}}\leq \mathrm{KS}$$

*holds true.*

Since below we directly refer to the main statement in [10], which assumes compactness, and for simplicity, the Theorem is formulated under this assumption, we however will discuss a relaxation of the assumption in Remark A1.

To prove the above corollary, one needs to verify that Conditions 1 and 2 are satisfied for one-dimensional systems if *T* is piecewise monotone. It is easy to see that Condition 2 holds true for *T* being aperiodic and ergodic: If *T* is aperiodic, for any ϵ>0, one can choose a finite ordered partition Q such that μ(Q)<ϵ holds true for all Q∈Q. The ergodicity then implies

$$\begin{array}{cc} \; & \sum_{Q\in Q}\underset{n\to \infty }{lim sup}\frac{1}{n}\sum_{l=1}^{n}\mu \left(Q\cap T^{-l}\left(Q\right)\right)=\sum_{Q\in Q}\mu {\left(Q\right)}^{2}<\sum_{Q\in Q}\mu \left(Q\right)\cdot \epsilon =\epsilon . \end{array}$$

One can also show that Condition 2 is true for non-ergodic aperiodic compact systems (see Remark A1 and [10]).

If *T* is (countably) piecewise monotone, there exists a finite (or countable infinite) ordered partition $M={\left\{M_{i}\right\}}_{i\in I}$ with H(M)<∞ satisfying (4), which is equivalent to

$$\mu^{2}\left(M_{i}\times M_{i}\cap R\cap {\left(T\times T\right)}^{-1}\left(R\right)\right)\in \left\{0,\mu^{2}\left(M_{i}\times M_{i}\cap {\left(T\times T\right)}^{-1}\left(R\right)\right)\right\}$$

for all i∈I. Therefore,

(7) $$\left\{M_{i}\times M_{i}\right\}\vee \left\{R,\Omega^{2}\backslash R\right\}\vee {\left(T\times T\right)}^{-1}\left(\{R,\Omega^{2}\backslash R\}\right)\overset{\left(4\right)}{≺}\left\{M_{i}\times M_{i}\right\}\vee {\left(T\times T\right)}^{-1}\left(\{R,\Omega^{2}\backslash R\}\right)$$

is true for all i∈I. Because M is an ordered partition, we have

(8) $$\left\{M_{i_{1}}\times M_{i_{2}}\right\}\vee \left\{R,\Omega^{2}\backslash R\right\}\overset{\left(3\right)}{≺}\left\{M_{i_{1}}\times M_{i_{2}}\right\}$$

for all $i_{1}\neq i_{2}\in I$. This implies

$$\begin{array}{cc} \; & \;M⊗M\vee \{R,\Omega^{2}\backslash R\}\\ = & \;\left(\underset{\begin{array}{c} \left(i_{1},i_{2}\right)\in I^{2}:\\ i_{1}\neq i_{2} \end{array}}{⋁}\left\{M_{i_{1}}\times M_{i_{2}}\right\}\vee \left\{R,\Omega^{2}\backslash R\right\}\right)\vee \left(\underset{i\in I}{⋁}\left\{M_{i}\times M_{i}\right\}\vee \left\{R,\Omega^{2}\backslash R\right\}\right)\\ \overset{\left(8\right)}{≺} & \;\underset{\begin{array}{c} \left(i_{1},i_{2}\right)\in I^{2}:\\ i_{1}\neq i_{2} \end{array}}{⋁}\left\{M_{i_{1}}\times M_{i_{2}}\right\}\vee \left(\underset{i\in I}{⋁}\left\{M_{i}\times M_{i}\right\}\vee \left\{R,\Omega^{2}\backslash R\right\}\right)\\ ≺ & \;\underset{\begin{array}{c} \left(i_{1},i_{2}\right)\in I^{2}:\\ i_{1}\neq i_{2} \end{array}}{⋁}\left\{M_{i_{1}}\times M_{i_{2}}\right\}\vee \left(\underset{i\in I}{⋁}\left\{M_{i}\times M_{i}\right\}\vee \left\{R,\Omega^{2}\backslash R\right\}\vee {\left(T\times T\right)}^{-1}\left(\left\{R,\Omega^{2}\backslash R\right\}\right)\right)\\ \overset{\left(7\right)}{≺} & \;\underset{\begin{array}{c} \left(i_{1},i_{2}\right)\in I^{2}:\\ i_{1}\neq i_{2} \end{array}}{⋁}\left\{M_{i_{1}}\times M_{i_{2}}\right\}\vee \left(\underset{i\in I}{⋁}\left\{M_{i}\times M_{i}\right\}{\left(T\times T\right)}^{-1}\left(\left\{R,\Omega^{2}\backslash R\right\}\right)\right)\\ ≺ & \;\underset{\left(i_{1},i_{2}\right)\in I^{2}}{⋁}\left\{M_{i_{1}}\times M_{i_{2}}\right\}{\left(T\times T\right)}^{-1}\left(\left\{R,\Omega^{2}\backslash R\right\}\right)\\ = & \;M⊗M\vee {\left(T\times T\right)}^{-1}\left(\left\{R,\Omega \backslash R\right\}\right). \end{array}$$

Hence, Condition 1 holds true if *T* is (countably) piecewise monotone. To show that Corollary 1 holds true if the dynamical system is not aperiodic, one splits the system into a periodic part and an aperiodic part in the following way:

Let

$$\Theta :=\overset{\infty }{\underset{t=1}{⋃}}\left\{\omega \in \Omega |T^{t}\left(\omega \right)=\omega \right\}$$

be the set of periodic points. Assume that μ(Θ)∉{0,1} is true. Then,

$$\begin{array}{cc} \bar{\mathrm{PE}}\leq & \;\underset{n\to \infty }{lim sup}\frac{1}{n}H\left(O\;P\left(n\right)\vee \left\{\Theta ,\Omega \backslash \Theta \right\}\right)\\ = & \;\underset{n\to \infty }{lim sup}\frac{1}{n}\left[H(O\;P\left(n\right)\vee \left\{\Theta ,\Omega \backslash \Theta \right\})-H\left(\Theta ,\Omega \backslash \Theta \right)\right] \end{array}$$

(9) $$\begin{array}{cc} = & \;\mu \left(\Theta \right)\cdot \underset{n\to \infty }{lim sup}\frac{1}{n}-\sum_{P_{\pi }\in O\;P\left(n\right)}\frac{\mu (P_{\pi }\cap \Theta )}{\mu (\Theta )}\log\left(\frac{\mu (P_{\pi }\cap \Theta )}{\mu (\Theta )}\right) \end{array}$$

(10) $$\begin{array}{cc} & +\mu \left(\Omega \backslash \Theta \right)\cdot \underset{n\to \infty }{lim sup}\frac{1}{n}-\sum_{P_{\pi }\in O\;P\left(n\right)}\frac{\mu (P_{\pi }\backslash \Theta )}{\mu (\Omega \backslash \Theta )}\log\left(\frac{\mu (P_{\pi }\backslash \Theta )}{\mu (\Omega \backslash \Theta )}\right) \end{array}$$

holds true, where (9) is the periodic part of the upper permutation entropy and (10) the aperiodic part. One can use the aperiodic version of Corollary 1 to show that the Kolmogorov–Sinai entropy is an upper bound for (10). The proof of Corollary 1 for non-aperiodic dynamical systems is complete with Lemma A5 in Appendix A.4, which shows that (9) is equal to 0.

### 3.2. Examples

In order to illustrate the discussion in Section 3.1, we consider two examples. The first one reflects the situation in Corollary 1, and the second one discusses the case m=2 in Condition 1 in Theorem 4.

**Example** **1** (Gaussian map)**.** *The map T:[0,1]→[0,1] with*

$$T\left(\omega \right)=\left\{\begin{array}{cc} 1/\omega \;\mathrm{mod}\;1, & \mathrm{if}\;\omega >0,\\ 0, & \mathrm{if}\;\omega =0 \end{array}\right.$$

**Figure 2:**
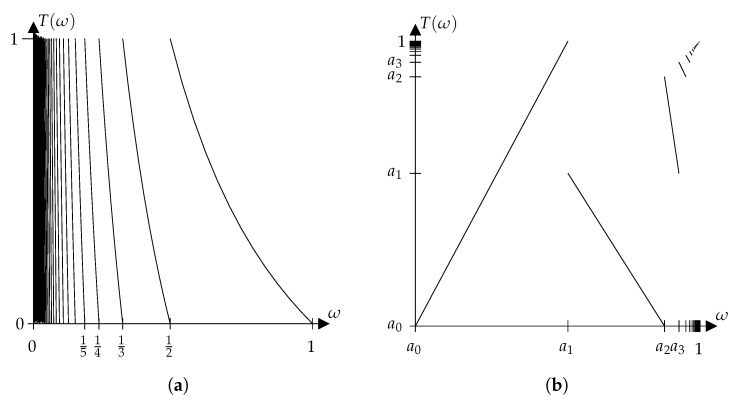
(**a**) Graph of the Gaussian map. (**b**) Graph of the map given in Example 2.

*is called a Gaussian map (see Figure 2a). This map is measure-preserving with regard to the measure μ, which is defined by $\mu \left(A\right)=\frac{1}{\log2}\int_{A}\frac{1}{1+x}\;dx$ for all A∈B [11]. The partition $M=\{[\frac{1}{n+1},\frac{1}{n}\left[|n\in N\right\}\cup \left\{\left\{0\right\}\right\}$ of [0,1] is a countably infinite partition into monotony intervals of T satisfying H(M)<∞. This map is countably piecewise monotone and ergodic. Thus, its Kolmogorov–Sinai entropy is equal to its permutation entropy.*

**Example** **2.** *Consider Ω=[0,1[ and the Borel σ-algebra B on* Ω*. Set*

$$\begin{array}{cc} S & :=\sum_{i=0}^{\infty }\frac{1}{\left(i+1\right){\left(\log\left(i+1\right)\right)}^{2}},\\ m_{i} & :=\frac{1}{S}\cdot \frac{1}{\left(i+1\right){\left(\log\left(i+1\right)\right)}^{2}}\;\mathrm{for}\;i\in N,\\ a_{0} & :=0,\\ a_{1} & :=m_{1},\\ a_{i} & :=1-\frac{m_{i+1}}{m_{1}}\;\mathrm{for}\;i\geq 2,\\ M_{i} & :=[a_{i-1},a_{i}]\;\mathrm{for}\;i\in N. \end{array}$$

*The map T:Ω→Ω is defined as piecewise linear on each set $M_{i}$ (see Figure 2b) by*

$$T\left(\omega \right)=\left\{\begin{array}{cc} \frac{\omega }{m_{1}}, & if\;\omega \in M_{1},\\ a_{i-1}\cdot \frac{a_{i}-\omega }{a_{i}-a_{i-1}}+a_{i-2}\cdot \frac{\omega -a_{i-1}}{a_{i}-a_{i-1}}, & if\;\omega \in M_{i}\;with\;i\in N\backslash \left\{1\right\}. \end{array}\right.$$

*Let λ be the one-dimensional Lebesgue measure. Define a measure μ on (Ω,B) by*

$$\mu \left(A\right):=\sum_{j=1}^{\infty }\frac{m_{j}\cdot \lambda \left(A\cap M_{j}\right)}{\lambda (M_{j})}$$

*for all A∈B.*

*One can verify that T is measure-preserving and ergodic with regard to μ. The partition $M:={\left\{M_{i}\right\}}_{i\in N}$ does satisfy (5) for m=1, but H(M)=∞ holds true. Therefore, Condition 1 does not hold true for m=1. However, one can show that Condition 1 holds true for m=2 and the partition $M^{\prime }:=\left\{M_{1},{⋃}_{m=2}^{\infty }M_{i}\right\}$, which implies that the Kolmogorov–Sinai entropy is equal to the permutation entropy of this map due to Theorem 4.*

## 4. A Supplementary Aspect

To determine under what conditions the Kolmogorov–Sinai entropy and the upper or lower permutation entropies coincide remains an open problem in the general case, and in the simple one-dimensional case of maps not being (countably) piecewise monotone the relation of Kolmogorov–Sinai and upper and lower permutation entropies is not completely understood. There is not even known an example where the entropies differ. Finally, we shortly want to discuss a further approach for discussing the relationship of Kolmogorov–Sinai entropy and upper and lower permutation entropies.

In [12], it was shown that under (1) the Kolmogorov–Sinai entropy is equal to the permutation entropy if roughly speaking the information contents of ‘words’ of *k* ’successive’ ordinal patterns of large length *n* is not too far from the information contents of ordinal patterns of length n+k−1. We want to explain this for the simple one-dimensional case and k=2.

The ordinal pattern of some $\left(x_{0},x_{1},\ldots ,x_{n}\right)$ contains all information on the order relation between the points $x_{0},x_{1},\ldots ,x_{n}$. When considering the ‘overlapping’ ordinal patterns of $\left(x_{0},x_{1},\ldots ,x_{n-1}\right)$ and $\left(x_{1},x_{2},\ldots ,x_{n}\right)$, one has the same information with one exception: The order relation between $x_{0}$ and $x_{n}$ is not known a priori. Looking at the related partitions, the missing information is quantified by the conditional entropy $H(O\;P\left(n+1\right)|O\;P\left(n\right)\vee T^{-1}\left(O\;P\left(n\right)\right))$. There is one situation reducing this missing information, namely that one of the $x_{i};i=1,2,\ldots ,n-1$ lies between $x_{0}$ and $x_{n}$. Then, the order relation between $x_{0}$ and $x_{n}$ is known by knowing the ordinal patterns of $\left(x_{0},x_{1},\ldots ,x_{n-1}\right)$ and $\left(x_{1},x_{2},\ldots ,x_{n}\right)$. Therefore, the following set is of some special interest:

(11) $$\begin{array}{cc} V_{n}:= & \left\{\omega \in \Omega |\omega \leq T^{n}\left(\omega \right)\;\mathrm{and}\;T^{s}\left(\omega \right)\notin \left(\omega ,T^{n}\left(\omega \right)\right)\;\mathrm{for}\;\mathrm{all}\;s\in \left\{1,2,\ldots ,n-1\right\}\right\}\\ \cup & \left\{\omega \in \Omega |T^{n}\left(\omega \right)\leq \omega \;\mathrm{and}\;T^{s}\left(\omega \right)\notin \left(T^{n}\left(\omega \right),\omega \right)\;\mathrm{for}\;\mathrm{all}\;s\in \left\{1,2,\ldots ,n-1\right\}\right\}. \end{array}$$

The following is shown in Appendix A.5:

**Lemma** **1.** *Let* Ω *be a subset of R and A=B be the Borel σ-algebra on* Ω*. Then,*

(12) $$H\left(O\;P\left(n+1\right)|O\;P\left(n\right)\vee T^{-1}\left(O\;P\left(n\right)\right)\right)\leq \log\left(2\right)\cdot \mu \left(V_{n}\right)$$

*holds true for all n∈N.*

This indicates that analyzing the measure of $V_{n}$ as defined in (11) can be a useful approach to gain inside into the relationship between different kinds of entropies based on ordinal patterns. In particular, the behavior of $\mu (V_{n})$ for n→∞ is of interest.

**Lemma** **2.** *Let* Ω *be a subset of R and A=B be the Borel σ-algebra on* Ω*. If T is ergodic, then*

$$\underset{n\to \infty }{lim inf}\mu \left(V_{n}\right)=0$$

*holds true, and, if (stronger) T is mixing, then*

$$\lim_{n\to \infty }\mu \left(V_{n}\right)=0$$

*holds true.*

The statement under the assumption of mixing has been shown in [5], and the proof in the ergodic case is given in Appendix A.5.

One can show that in the simple one-dimensional case the Kolmogorov–Sinai entropy is equal to the permutation entropy if

(13) $$\sum_{n=1}^{\infty }H\left(O\;P\left(n+1\right)|O\;P\left(n\right)\vee T^{-1}\left(O\;P\left(n\right)\right)\right)<\infty$$

holds true. Using (12), this is the case when $\sum_{n=1}^{\infty }\mu \left(V_{n}\right)$ is finite, providing a fast decay of the $\mu (V_{n})$. However, we have $\sum_{n=1}^{\infty }\mu \left(V_{n}\right)=\infty$ as stated in Theorem 5, which will be proved in Appendix A.6.

**Theorem** **5.** *Let* Ω *be a subset of R, A=B be the Borel σ-algebra on* Ω *and T be aperiodic and ergodic. Then,*

$$\sum_{n=1}^{\infty }\mu \left(V_{n}\right)=\infty$$

*holds true.*

Although formula $\sum_{n=1}^{\infty }\mu \left(V_{n}\right)<\infty$ is false, we cannot answer the question of whether or when (13) is valid. Possibly, an answer to this question, and a better understanding of the kind of decay of the $\mu (V_{n})$, could be helpful in further investigating the relationship of Kolmogorov–Sinai entropy and upper and lower permutation entropies, at least in the simple one-dimensional ergodic case.

## 5. Conclusions

With Theorem 1, we have slightly generalized a statement given in [3] by removing a technical assumption and using more basic combinatorial arguments. The remaining assumption (1) on the random vector X cannot be weakened in general.

In Section 2.3, we have shown that the equality of the permutation entropy and the Kolmogorov–Sinai entropy implies the equality of conditional permutation entropy and Kolmogorov–Sinai entropies as well. We considered two different kinds of conditional permutation entropy, which have turned out to be equal in the cases considered in Section 2.3; it is however not clear whether these two kinds of conditional permutation entropy are equal in the general.

In Section 4, we have established some condition under which these two kinds of conditional entropy are equal, independently from of the equality between permutation and Kolmogorov–Sinai entropies. This condition is based on a concept introduced in [5] that was originally introduced as a tool for better understanding the relationship between permutation and Kolmogorov–Sinai entropies in a general setting. However, with Theorem 5, we have shown that this tool cannot directly be used to show the equality between permutation and Kolmogorov–Sinai entropies. It is an interesting question of whether and how a clever adaption and improvement of it can allow for new insights in the relationship between permutation and Kolmogorov–Sinai entropies.

In Section 3, we considered the simpler one-dimensional case. With Theorem 4, we have given two conditions under which the permutation entropy is a lower bound for the Kolmogorov–Sinai entropy. This theorem generalizes previous statements in [1] and slightly generalizes a statement in [10]. One of the conditions (Condition 2) holds true for a large class of dynamical systems, while, for the other one (Condition 1) to hold true, it is necessary that the system is in some sense ’order preserving’. It is still an unsolved and interesting question, whether Condition 1 can be weakened, especially since, to the best of our knowledge, there does not exist a counterexample to the equality of permutation entropy and Kolmogorov–Sinai entropies. Finding a generalization of Theorem 4 to a multidimensional setting is a further interesting question one could ask.

## Appendix A. Proofs

### Appendix A.1. Preliminaries

Given a probability space (Ω,A,μ) and two σ-algebras $A_{1},A_{2}\subseteq A$, we write

$$A_{1}\overset{\mu }{\subseteq }A_{2}$$

if for all $A_{1}\in A_{1}$ there exists $A_{2}\in A_{2}$ with $\mu \left(A_{1}\backslash A_{2}\right)+\mu \left(A_{2}\backslash A_{1}\right)=0$. We write

$$A_{1}\overset{\mu }{=}A_{2}$$

if both $A_{1}\overset{\mu }{\subseteq }A_{2}$ and $A_{2}\overset{\mu }{\subseteq }A_{1}$ hold true.

For a collection of R-valued random variables ${\left\{X_{i}\right\}}_{i\in I}$ defined on some measure space (Ω,A), we denote by

$$\sigma \left(\{X_{i}|i\in I\}\right)$$

the smallest σ-algebra containing all sets in $\left\{X_{i}^{-1}\left(B\right)|i\in I,B\in B\right\}$, where B is the Borel σ-algebra on R.

Given a family of disjoint sets P⊆A and some set Q∈A, we define

Δ(P|Q):={P∈P∣μ(P∩Q)>0}

as those sets in P that are intersecting *Q* almost surely.

For two partitions P and Q of Ω in A, the number of elements in Δ(P|Q) can be used for an upper bound of the conditional entropy H(P|Q):=H(P∨Q)−H(Q):

Consider the function f:[0,1]→R with f(x)=xlog(x). Since *f* is convex, Jensen’s inequality provides

$$\begin{array}{cc} \; & \sum_{P\in \Delta (P|Q)}\mu \left(P\cap Q\right)\log\left(\mu \left(P\cap Q\right)\right)\\ = & \#\Delta \left(P|Q\right)\sum_{P\in \Delta (P|Q)}\frac{1}{\#\Delta (P|Q)}\cdot f\left(\mu \left(P\cap Q\right)\right)\\ \geq & \#\Delta \left(P|Q\right)\cdot f\left(\sum_{P\in \Delta (P|Q)}\frac{1}{\#\Delta (P|Q)}\cdot \mu \left(P\cap Q\right)\right)\\ = & \#\Delta \left(P|Q\right)\cdot f\left(\frac{\mu (Q)}{\#\Delta (P|Q)}\right)\\ = & \#\Delta \left(P|Q\right)\cdot \frac{\mu (Q)}{\#\Delta (P|Q)}\cdot \log\left(\frac{\mu (Q)}{\#\Delta (P|Q)}\right)\\ = & \mu \left(Q\right)\cdot \left(\log(\mu (Q))-\log(\#\Delta (P|Q))\right) \end{array}$$

for all Q∈Q. Using the above inequality implies

$$\begin{array}{cc} H(P\vee Q) & =-\sum_{Q\in Q}\sum_{P\in P}\mu \left(P\cap Q\right)\log\left(\mu \left(P\cap Q\right)\right)\\ \; & =-\sum_{Q\in Q}\sum_{P\in \Delta (P|Q)}\mu \left(P\cap Q\right)\log\left(\mu \left(P\cap Q\right)\right)\\ \; & \leq -\sum_{Q\in Q}\mu \left(Q\right)\cdot \left(\log(\mu (Q))-\log(\#\Delta (P|Q))\right)\\ \; & =H\left(Q\right)+\sum_{Q\in Q}\mu \left(Q\right)\cdot \log\left(\#\Delta \left(P|Q\right)\right). \end{array}$$

This is equivalent to

(A1) $$H\left(P|Q\right)\leq \sum_{Q\in Q}\mu \left(Q\right)\cdot \log\left(\#\Delta \left(P|Q\right)\right).$$

### Appendix A.2. Proof of the Equality in Formula (2)

The proof is based on the following Lemma A1 and Corollary A1.

**Lemma** **A1.**
*Let X:Ω→R be a random variable and U a finite ordered partition of R with regard to the image measure $\mu_{X}$. Then, for $P:=X^{-1}\left(U\right)$, n∈N and all $P_{\pi }\in O\;P^{X}\left(n\right)$*

#Δ(P(n)|Pπ)≤n+#U−1#U−1

*holds true.*

**Proof.** Set I={1,2,…,#U} and label the sets $U_{i}\in U$ with i∈I in such a way that

(A2) $$i_{1}<i_{2}\Rightarrow \mu^{2}\left(\left\{\left(\omega_{1},\omega_{2}\right)\in \left(X^{-1}\left(U_{i_{1}}\right)\times X^{-1}\left(U_{i_{2}}\right)\right):X\left(\omega_{1}\right)>X\left(\omega_{2}\right)\right\}\right)=0$$

holds true for all $i_{1},i_{2}\in I$. Since U is assumed to be an ordered partition, this is always possible. Set $P_{i}:=X^{-1}\left(U_{i}\right)$ for all i∈I so that $P={\left\{P_{i}\right\}}_{i\in I}$. Fix n∈N and $P_{\pi }\in O\;P^{X}\left(n\right)$. Using

$$P\left(i\right)=\overset{n-1}{\underset{t=0}{⋂}}T^{-t}\left(P_{i_{t}}\right)$$

for all $i=\left(i_{0},i_{1},\ldots i_{n-1}\right)\in I^{n}$, we have

$$\#\Delta \left(P^{\left(n\right)}|P_{\pi }\right)=\#\left\{i\in I^{n}:\mu \left(P\left(i\right)\cap P_{\pi }\right)>0\right\}.$$

There exists a permutation $\pi =(r_{0},r_{1},\ldots ,r_{n-1})$ of {0,1,…,n−1} such that

$$X\left(T^{r_{0}}\left(\omega \right)\right)\leq X\left(T^{r_{1}}\left(\omega \right)\right)\leq \ldots \leq X\left(T^{r_{n-1}}\left(\omega \right)\right)$$

holds true for all $\omega \in P_{\pi }$. Using (A2), this implies

$$i_{r_{0}}\leq i_{r_{1}}\leq \ldots \leq i_{r_{n-1}}$$

for all $i=\left(i_{0},i_{1},\ldots i_{n-1}\right)\in I^{n}$ with $\mu (P\left(i\right)\cap P_{\pi })>0$. Therefore,

#Δ(P(n)|Pπ)=#{(i0,i1,…in−1)∈In:μ(P(i)∩Pπ)>0}≤#{(i0,i1,…in−1)∈In:ir0≤ir1≤…≤irn−1}=n+#U−1#U−1

holds true for all $P_{\pi }\in O\;P^{X}\left(n\right)$. □

The lemma can be used to directly prove the following result.

**Corollary** **A1.**
*Let $X=\left(X_{1},X_{2},\ldots ,X_{d}\right):\Omega \to R^{d}$ be a random vector and U a finite partition of R into intervals. Then,*

$$h\left(\overset{d}{\underset{i=1}{⋁}}X_{i}^{-1}\left(U\right)\right)\leq \lim_{k\to \infty }h\left(O\;P^{X}\left(k\right)\right)$$

*holds true.*

**Proof.** Take k,m∈N and set $P_{i}:=X_{i}^{-1}\left(U\right)$. Then,

$$\begin{array}{cc} \; & \;H(P_{i}^{\left(mk\right)}|{\left(O\;P^{X_{i}}\left(k\right)\right)}^{\left(mk\right)})\\ \leq & \;\sum_{t=0}^{m-1}H\left(T^{-kt}\left(P_{i}^{\left(k\right)}\right)|{\left(O\;P^{X_{i}}\left(k\right)\right)}^{\left(mk\right)}\right)\\ \leq & \;\sum_{t=0}^{m-1}H\left(T^{-kt}\left(P_{i}^{\left(k\right)}\right)|T^{-kt}\left(O\;P^{X_{i}}\left(k\right)\right)\right)\\ = & \;mH(P_{i}^{\left(k\right)}|O\;P^{X_{i}}\left(k\right)) \end{array}$$

holds true for all i∈{1,2,…,d}. Together with (A1) and Lemma A1, this provides

$$\begin{array}{cc} h\left(\overset{d}{\underset{i=1}{⋁}}P_{i}\right) & =\lim_{n\to \infty }\frac{1}{n}H\left(\overset{d}{\underset{i=1}{⋁}}P_{i}^{\left(n\right)}\right)=\lim_{m\to \infty }\frac{1}{mk}H\left(\overset{d}{\underset{i=1}{⋁}}P_{i}^{\left(mk\right)}\right)\\ \; & \leq \lim_{m\to \infty }\frac{1}{mk}H\left(\overset{d}{\underset{i=1}{⋁}}P_{i}^{\left(mk\right)}\vee {\left(O\;P^{X}\left(k\right)\right)}^{\left(mk\right)}\right)\\ \; & =\lim_{m\to \infty }\frac{1}{mk}\left[H\left({\left(O\;P^{X}\left(k\right)\right)}^{\left(mk\right)}\right)+H\left(\overset{d}{\underset{i=1}{⋁}}P_{i}^{\left(mk\right)}|{\left(O\;P^{X}\left(k\right)\right)}^{\left(mk\right)}\right)\right]\\ \; & \leq \lim_{m\to \infty }\frac{1}{mk}\left[H\left({\left(O\;P^{X}\left(k\right)\right)}^{\left(mk\right)}\right)+\sum_{i=1}^{d}H\left(P_{i}^{\left(mk\right)}|{\left(O\;P^{X}\left(k\right)\right)}^{\left(mk\right)}\right)\right] \end{array}$$

≤limm→∞1mkH((OPX(k))(mk))+∑i=1dHPi(mk)|(OPXi(k))(mk)≤limm→∞1mkH((OPX(k))(mk))+∑i=1d1kH(Pi(k)|OPXi(k))=h(OPX(k))+∑i=1d1kH(Pi(k)|OPXi(k))≤h(OPX(k))+∑i=1d1k∑Pπ∈OPXi(k)μ(Pπ)log(#Δ(Pi(k)|Pπ))≤h(OPX(k))+∑i=1d1k∑Pπ∈OPXi(k)μ(Pπ)logk+#U−1#U−1≤h(OP(k))+dklog(k+#U−1)#U−1=h(OP(k))+d(#U−1)·log(k+#U−1)k

for all k∈N, which implies

$$\begin{array}{cc} h(P) & \leq \lim_{k\to \infty }\left[h\left(T,O\;P\left(k\right)\right)+d\left(\#U-1\right)\cdot \frac{\log(k+\#U-1)}{k}\right]=\lim_{k\to \infty }h\left(O\;P\left(k\right)\right). \end{array}$$

 □

We are now able to prove of the equality in (2). Let $p_{i}:R^{d}\to R$ with $p_{i}\left(\left(x_{1},x_{2},\ldots ,x_{d}\right)\right)=x_{i}$ be the projection on the *i*-th coordinate, and let $B(R^{d})$ denote the Borel σ-algebra on $R^{d}$. Since this σ-algebra is generated by sets of the type

$$I_{1}\times I_{2}\times \ldots I_{d},$$

where $I_{i}$ are intervals, there exists an increasing sequence of finite partition ${\left(U_{l}\right)}_{l\in N}$ of R into intervals, such that

$$B\left(R^{d}\right)=\sigma \left(\left\{p_{i}^{-1}\left(U_{l}\right)|l\in N,i\in \left\{1,2,\ldots ,d\right\}\right\}\right)$$

holds true. Using (1), this implies

$$\begin{array}{cc} A & =\sigma \left(\left\{T^{-t}\left(X^{-1}\left(p_{i}^{-1}\left(U_{l}\right)\right)\right)|t\in N_{0},l\in N,i\in \left\{1,2,\ldots ,d\right\}\right\}\right)\\ \; & =\sigma \left(\left\{T^{-t}\left(X_{i}^{-1}\left(U_{l}\right)\right)|t\in N_{0},l\in N,i\in \left\{1,2,\ldots ,d\right\}\right\}\right)\\ \; & =\sigma \left(\left\{T^{-t}\left(\overset{d}{\underset{i=1}{⋁}}X_{i}^{-1}\left(U_{l}\right)\right)|t\in N_{0},l\in N\}\right\}\right). \end{array}$$

Thus, ${\left(P_{l}\right)}_{l\in N}$ with

$$P_{l}:=\overset{d}{\underset{i=1}{⋁}}X_{i}^{-1}\left(U_{l}\right)$$

is a generating sequence of finite partitions, which implies (see e.g., [13])

$$\mathrm{KS}=\lim_{l\to \infty }h\left(P_{l}\right).$$

Corollary A1 provides

$$h\left(P_{l}\right)\leq \lim_{k\to \infty }h\left(O\;P^{X}\left(k\right)\right)$$

for all l∈N. Combining the two previous statements yields

(A3) $$\mathrm{KS}=\lim_{l\to \infty }h\left(P_{l}\right)\leq \lim_{k\to \infty }h\left(O\;P^{X}\left(k\right)\right).$$

On the other hand,

$$\mathrm{KS}=\underset{P}{\sup}h\left(P\right)\geq \lim_{k\to \infty }h\left(O\;P^{X}\left(k\right)\right)$$

holds true, which, together with (A3), finishes the proof of the equality in (2).

### Appendix A.3. Proof of Theorem 2

For preparing the proof of Theorem 2, let us first give two lemmata.

**Lemma** **A2.** *Let ${\left(P_{n}\right)}_{n\in N}$ be a sequence of finite partitions of* Ω *in A satisfying*

(A4) $$P_{n}\vee T^{-1}\left(P_{n}\right)≺P_{n+1}.$$

*Then,*

$$\underset{n\to \infty }{lim inf}H\left(P_{n}\left|T^{-1}\left(P_{n}^{\left(k\right)}\right)\right.\right)\leq \underset{n\to \infty }{lim inf}H\left(P_{n+1}^{\left(k\right)}\left|P_{n}^{\left(k\right)}\right.\right)\leq \underset{n\to \infty }{lim inf}\frac{1}{n}H\left(P_{n}\right)$$

*holds true for all k∈N.*

**Proof.** Take k∈N. We have

$$\begin{array}{cc} \; & \underset{n\to \infty }{lim inf}H\left(P_{n}\left|T^{-1}\left(P_{n}^{\left(k\right)}\right)\right.\right)=\underset{n\to \infty }{lim inf}\left[H\left(P_{n}^{\left(k+1\right)}\right)-H\left(T^{-1}\left(P_{n}^{\left(k\right)}\right)\right)\right]\\ = & \;\underset{n\to \infty }{lim inf}\left[H\left(P_{n}^{\left(k+1\right)}\right)-H\left(P_{n}^{\left(k\right)}\right)\right]\overset{\left(A4\right)}{\leq }\underset{n\to \infty }{lim inf}\left[H\left(P_{n+1}^{\left(k\right)}\right)-H\left(P_{n}^{\left(k\right)}\right)\right]=\underset{n\to \infty }{lim inf}H\left(P_{n+1}^{\left(k\right)}\left|P_{n}^{\left(k\right)}\right.\right). \end{array}$$

The Stolz–Cesàro theorem further provides

$$\begin{array}{cc} \; & \;\underset{n\to \infty }{lim inf}H\left(P_{n+1}^{\left(k\right)}\left|P_{n}^{\left(k\right)}\right.\right)\leq \underset{n\to \infty }{lim inf}\frac{1}{n}\sum_{i=1}^{n}\left[H\left(P_{i+1}^{\left(k\right)}\left|P_{i}^{\left(k\right)}\right.\right)\right]\\ = & \;\underset{n\to \infty }{lim inf}\frac{1}{n}\sum_{i=1}^{n}\left[H\left(P_{i+1}^{\left(k\right)}\right)-H\left(P_{i}^{\left(k\right)}\right)\right]=\underset{n\to \infty }{lim inf}\frac{1}{n}\left[H\left(P_{n+1}^{\left(k\right)}\right)-H\left(P_{1}^{\left(k\right)}\right)\right]\\ = & \;\underset{n\to \infty }{lim inf}\frac{1}{n}H\left(P_{n+1}^{\left(k\right)}\right)\overset{\left(A4\right)}{\leq }\underset{n\to \infty }{lim inf}\frac{1}{n}H\left(P_{n+k}\right)=\underset{n\to \infty }{lim inf}\frac{1}{n}H\left(P_{n}\right). \end{array}$$

 □

Notice that (A4) is fulfilled for $P_{n}:=O\;P^{X}\left(n\right)$.

**Lemma** **A3.**
*Let $X=\left(X_{1},X_{2},\ldots ,X_{d}\right):\Omega \to R^{d}$ be a random vector satisfying (1). Then,*

$$\mathrm{KS}\leq \underset{n\to \infty }{lim inf}H\left(O\;P^{X}\left(n\right)\left|T^{-1}\left({\left(O\;P^{X}\left(n\right)\right)}^{\left(k\right)}\right)\right.\right)$$

*holds true for all k∈N.*

**Proof.** According to Theorem 1, we have

$$\mathrm{KS}=\lim_{n\to \infty }h\left(T,O\;P^{X}\left(n\right)\right).$$

Using the future formula for the entropy rate (see e.g., [13]), we can write

$$h\left(O\;P^{X}\left(n\right)\right)=\lim_{l\to \infty }H\left(O\;P^{X}\left(n\right)\left|T^{-1}\left({\left(O\;P^{X}\left(n\right)\right)}^{\left(l\right)}\right)\right.\right)$$

for all n∈N. This implies

$$\begin{array}{cc} \mathrm{KS} & =\lim_{n\to \infty }\lim_{l\to \infty }H\left(O\;P^{X}\left(n\right)\left|T^{-1}\left({\left(O\;P^{X}\left(n\right)\right)}^{\left(l\right)}\right)\right.\right)\\ \; & \leq \underset{n\to \infty }{lim inf}H\left(O\;P^{X}\left(n\right)\left|T^{-1}\left({\left(O\;P^{X}\left(n\right)\right)}^{\left(k\right)}\right)\right.\right) \end{array}$$

for all k∈N. □

Now, Lemmas A2 and A3 provide

$$\begin{array}{cc} \mathrm{KS} & \leq \underset{n\to \infty }{lim inf}H\left(O\;P^{X}\left(n\right)\left|T^{-1}\left({\left(O\;P^{X}\left(n\right)\right)}^{\left(k\right)}\right)\right.\right)\\ \; & \leq \underset{n\to \infty }{lim inf}H\left({\left(O\;P^{X}\left(n+1\right)\right)}^{\left(k\right)}\left|{\left(O\;P^{X}\left(n\right)\right)}^{\left(k\right)}\right.\right)\\ \; & \leq {\underline{\mathrm{PE}}}^{X}\leq {\bar{\mathrm{PE}}}^{X}. \end{array}$$

The assumption $\mathrm{KS}\geq {\bar{\mathrm{PE}}}^{X}$ then implies

$$\begin{array}{cc} \mathrm{KS} & =\underset{n\to \infty }{lim inf}H\left(O\;P^{X}\left(n\right)\left|T^{-1}\left({\left(O\;P^{X}\left(n\right)\right)}^{\left(k\right)}\right)\right.\right)\\ \; & =\underset{n\to \infty }{lim inf}H\left({\left(O\;P^{X}\left(n+1\right)\right)}^{\left(k\right)}\left|{\left(O\;P^{X}\left(n\right)\right)}^{\left(k\right)}\right.\right)\\ \; & ={\underline{\mathrm{PE}}}^{X}={\bar{\mathrm{PE}}}^{X} \end{array}$$

for all k∈N.

### Appendix A.4. Proofs for the Simple One-Dimensional Case

This subsection is mainly devoted to the proofs of Theorem 4 and to the proof of Lemma A5 mentioned at the end of Section 3.1. Recall the assumption that Ω is a subset of R and A=B the Borel σ-algebra on Ω. The following lemma is a step to the proof of Theorem 4.

**Lemma** **A4.** *Let* Ω *be a subset of R and A=B be the Borel σ-algebra on* Ω*. Suppose, there exist an ordered partition $M={\left\{M_{i}\right\}}_{i\in I}$ and an m∈N satisfying (5). Then, for all n∈N with n≥m and multiindices $i=\left(i_{0},i_{1},\ldots ,i_{n-1}\right)\in I^{n}$*

$$\#\Delta \left(O\;P\left(n\right)|M\left(i\right)\right)\leq 2^{\sum_{u=1}^{m}\#\left\{s\in \left\{0,1,\ldots ,n-1\right\}|i_{s}=i_{n-u}\;\mathrm{and}\;s\neq n-u\right\}}$$

*holds true.*

**Proof.** Fix m∈N and n∈N with n≥m and $i=\left(i_{0},i_{1},\ldots ,i_{n-1}\right)\in I^{n}$. We will show that

(A5) $$\begin{array}{cc} \; & \;M^{\left(s\right)}⊗M^{\left(s\right)}\vee \overset{s-1}{\underset{t=0}{⋁}}{\left(T\times T\right)}^{-t}\left(\left\{R,\Omega^{2}\backslash R\right\}\right)\\ ≺ & \;M^{\left(s\right)}⊗M^{\left(s\right)}\vee \overset{s-1}{\underset{t=s-m}{⋁}}{\left(T\times T\right)}^{-t}\left(\left\{R,\Omega^{2}\backslash R\right\}\right) \end{array}$$

holds true for all s∈N with s≥m using induction over *s*: The above statement is trivial for s=m. Suppose that (A5) holds true for some s∈N with s≥m. We will show that (A5) then holds true for s+1:

(A6) $$\begin{array}{cc} \; & \;M^{\left(s+1\right)}⊗M^{\left(s+1\right)}\vee \overset{s}{\underset{t=0}{⋁}}{\left(T\times T\right)}^{-t}\left(\{R,\Omega^{2}\backslash R\}\right)\\ = & \;{\left(T\times T\right)}^{-1}\left(M^{\left(s\right)}⊗M^{\left(s\right)}\vee \overset{s-1}{\underset{t=0}{⋁}}{\left(T\times T\right)}^{-t}\left(\{R,\Omega^{2}\backslash R\}\right)\right)\\ \; & \vee M^{\left(m\right)}⊗M^{\left(m\right)}\vee \left\{R,\Omega^{2}\backslash R\right\}\\ \overset{\left(5\right)}{≺} & \;{\left(T\times T\right)}^{-1}\left(M^{\left(s\right)}⊗M^{\left(s\right)}\vee \overset{s-1}{\underset{t=0}{⋁}}{\left(T\times T\right)}^{-t}\left(\{R,\Omega^{2}\backslash R\}\right)\right)\\ \; & \vee M^{\left(m\right)}⊗M^{\left(m\right)}\vee \overset{m}{\underset{u=1}{⋁}}{\left(T\times T\right)}^{-u}\left(\{R,\Omega^{2}\backslash R\}\right)\\ = & \;{\left(T\times T\right)}^{-1}\left(M^{\left(s\right)}⊗M^{\left(s\right)}\vee \overset{s-1}{\underset{t=0}{⋁}}{\left(T\times T\right)}^{-t}\left(\{R,\Omega^{2}\backslash R\}\right)\right)\vee M⊗M\\ ≺ & \;{\left(T\times T\right)}^{-1}\left(M^{\left(s\right)}⊗M^{\left(s\right)}\vee \overset{s-1}{\underset{t=s-m}{⋁}}{\left(T\times T\right)}^{-t}\left(\{R,\Omega^{2}\backslash R\}\right)\right)\vee M⊗M\\ = & \;M^{\left(s+1\right)}⊗M^{\left(s+1\right)}\vee \overset{s}{\underset{t=s+1-m}{⋁}}{\left(T\times T\right)}^{-t}\left(\{R,\Omega^{2}\backslash R\}\right). \end{array}$$

In (A6), the induction hypotheses was used. Notice that

$$\begin{array}{cc} M^{\left(n\right)} & =\overset{n}{\underset{s=1}{⋁}}{\left(id,T\right)}^{-n+s}\left(M^{\left(s\right)}⊗M^{\left(s\right)}\right),\\ O\;P\left(n\right) & =\overset{n}{\underset{s=1}{⋁}}{\left(id,T\right)}^{-n+s}\left(\overset{s-1}{\underset{t=0}{⋁}}{\left(T\times T\right)}^{-t}\left(\left\{R,\Omega^{2}\backslash R\right\}\right)\right) \end{array}$$

hold true. This implies

$$\begin{array}{cc} M^{\left(n\right)}\vee O\;P\left(n\right)= & \;\overset{n}{\underset{s=1}{⋁}}{\left(id,T\right)}^{-n+s}\left(M^{\left(s\right)}⊗M^{\left(s\right)}\vee \overset{s-1}{\underset{t=0}{⋁}}{\left(T\times T\right)}^{-t}\left(\left\{R,\Omega^{2}\backslash R\right\}\right)\right)\\ = & \;\overset{m-1}{\underset{s=1}{⋁}}{\left(id,T\right)}^{-n+s}\left(M^{\left(s\right)}⊗M^{\left(s\right)}\vee \overset{s-1}{\underset{t=0}{⋁}}{\left(T\times T\right)}^{-t}\left(\left\{R,\Omega^{2}\backslash R\right\}\right)\right)\\ \; & \vee \overset{n}{\underset{s=m}{⋁}}{\left(id,T\right)}^{-n+s}\left(M^{\left(s\right)}⊗M^{\left(s\right)}\vee \overset{s-1}{\underset{t=0}{⋁}}{\left(T\times T\right)}^{-t}\left(\left\{R,\Omega^{2}\backslash R\right\}\right)\right)\\ \overset{\left(A5\right)}{≺} & \;\overset{m-1}{\underset{s=1}{⋁}}{\left(id,T\right)}^{-n+s}\left(M^{\left(s\right)}⊗M^{\left(s\right)}\vee \overset{s-1}{\underset{t=0}{⋁}}{\left(T\times T\right)}^{-t}\left(\left\{R,\Omega^{2}\backslash R\right\}\right)\right)\\ & \vee \overset{n}{\underset{s=m}{⋁}}{\left(id,T\right)}^{-n+s}\left(M^{\left(s\right)}⊗M^{\left(s\right)}\vee \overset{s-1}{\underset{t=s-m}{⋁}}{\left(T\times T\right)}^{-t}\left(\left\{R,\Omega^{2}\backslash R\right\}\right)\right)\\ = & \;M^{\left(n\right)}\vee \overset{n-1}{\underset{s=0}{⋁}}\overset{n-1}{\underset{t=n-m}{⋁}}{\left(T^{s},T^{t}\right)}^{-1}\left(\left\{R,\Omega^{2}\backslash R\right\}\right). \end{array}$$

Therefore,

(A7) $$\begin{array}{cc} \#\Delta (O\;P\left(n\right)|M\left(i\right)) & =\#\Delta (M^{\left(n\right)}\vee O\;P\left(n\right)|M\left(i\right))\\ \; & \leq \#\Delta \left(M^{\left(n\right)}\vee \overset{n-1}{\underset{s=0}{⋁}}\overset{n-1}{\underset{t=n-m}{⋁}}{\left(T^{s},T^{t}\right)}^{-1}\left(\left\{R,\Omega^{2}\backslash R\right\}\right)|M\left(i\right)\right)\\ \; & =\#\Delta \left(\overset{n-1}{\underset{s=0}{⋁}}\overset{n-1}{\underset{t=n-m}{⋁}}{\left(T^{s},T^{t}\right)}^{-1}\left(\left\{R,\Omega^{2}\backslash R\right\}\right)|M\left(i\right)\right)\\ \; & \leq \prod_{s=0}^{n-1}\prod_{t=n-m}^{n-1}\#\Delta \left({\left(T^{s},T^{t}\right)}^{-1}\left(\left\{R,\Omega^{2}\backslash R\right\}\right)|M\left(i\right)\right) \end{array}$$

holds true. Notice that

$${\left(T^{s},T^{t}\right)}^{-1}\left(\left\{R,\Omega^{2}\backslash R\right\}\right)=\left\{\left\{\omega \in \Omega :T^{s}\left(\omega \right)<T^{t}\left(\omega \right)\right\},\left\{\omega \in \Omega :T^{s}\left(\omega \right)\geq T^{t}\left(\omega \right)\right\}\right\}.$$

For s=t, we have

$$\#\Delta \left({\left(T^{s},T^{t}\right)}^{-1}\left(\left\{R,\Omega^{2}\backslash R\right\}\right)|M\left(i\right)\right)=\#\Delta \left(\{\emptyset ,\Omega \}|M(i)\right)=1.$$

If $i_{s}\neq i_{t}$ is true, using the fact that M is an ordered partition yields

$$\begin{array}{cc} \; & \;\#\Delta \left({\left(T^{s},T^{t}\right)}^{-1}\left(\left\{R,\Omega^{2}\backslash R\right\}\right)|M\left(i\right)\right)\\ = & \;\#\Delta \left({\left(T^{s},T^{t}\right)}^{-1}\left(\left(M_{i_{s}}\times M_{i_{t}}\right)\vee \left\{R,\Omega^{2}\backslash R\right\}\right)|M\left(i\right)\right)\\ \overset{\left(3\right)}{=} & \;\#\Delta \left({\left(T^{s},T^{t}\right)}^{-1}\left(M_{i_{s}}\times M_{i_{t}}\right)|M\left(i\right)\right)=1. \end{array}$$

For all other cases, we have

$$\#\Delta \left({\left(T^{s},T^{t}\right)}^{-1}\left(\left\{R,\Omega^{2}\backslash R\right\}\right)|M\left(i\right)\right)\leq \#\left({\left(T^{s},T^{t}\right)}^{-1}\left(\left\{R,\Omega^{2}\backslash R\right\}\right)\right)=2.$$

The above observations can be summarized as

$$\#\Delta \left({\left(T^{s},T^{t}\right)}^{-1}\left(\left\{R,\Omega^{2}\backslash R\right\}\right)|M\left(i\right)\right)\left\{\begin{array}{cc} =1 & \mathrm{if}\;s=t\;\mathrm{or}\;i_{s}\neq i_{t},\\ \leq 2 & \mathrm{if}\;s\neq t\;\mathrm{and}\;i_{s}=i_{t}. \end{array}\right.$$

In combination with (A7), this provides

$$\begin{array}{cc} \; & \;\#\Delta \left({\left(T^{s},T^{t}\right)}^{-1}\left(\left\{R,\Omega^{2}\backslash R\right\}\right)|M\left(i\right)\right)\\ \leq & \;2^{\sum_{t=n-m}^{n-1}\#\{s\in \left\{0,1,\ldots ,n-1|i_{s}=i_{t}\;\mathrm{and}\;s\neq t\right\}}\\ = & \;2^{\sum_{u=1}^{m}\#\{s\in \left\{0,1,\ldots ,n-1|i_{s}=i_{n-u}\;\mathrm{and}\;s\neq n-u\right\}}. \end{array}$$

 □

Notice that the above Lemma immediately implies

(A8) $$\begin{array}{cc} H(O\;P\left(n\right)) & \leq H\left(O\;P\left(n\right)\vee M^{\left(n\right)}\right)=H\left(M^{\left(n\right)}\right)+H\left(O\;P\left(n\right)|M^{\left(n\right)}\right)\\ \; & \overset{\left(A1\right)}{\leq }H\left(M^{\left(n\right)}\right)+\sum_{i\in I^{n}}\mu \left(M\left(i\right)\right)\cdot \log\left(\#\Delta \left(O\;P\left(n\right)|M\left(i\right)\right)\right)\\ \; & \leq H\left(M^{\left(n\right)}\right)+\log\left(2\right)\cdot nm. \end{array}$$

We come now to the proof of Theorem 4, which slightly generalizes a proof given in [10], where the case m=1 was considered. For better readability, we restate this proof with the generalization to arbitrary m∈N at the appropriate places within the proof.

Take ϵ>0. According to Conditions 1 and 2, there exist finite or countably infinite ordered partitions $M={\left\{M_{i}\right\}}_{i\in I}$, $Q={\left\{Q_{j}\right\}}_{j\in J}$ and m∈N with H(M)<∞ and H(Q)<∞ satisfying (5) and (6). Consider the partition

$$P:=M\vee Q={\left\{M_{i}\cap Q_{j}\right\}}_{(i,j)\in I\times J}=:{\left\{P_{\left(i,j\right)}\right\}}_{(i,j)\in I\times J}.$$

Notice that P is again a finite or countably infinite ordered partition with H(P)<H(M)+H(Q)<∞. Using (A1), this implies

(A9) $$\begin{array}{cc} \bar{\mathrm{PE}} & \leq \underset{n\to \infty }{lim sup}\frac{1}{n}H\left(O\;P\left(n\right)\vee P^{\left(n\right)}\right)\\ \; & =h\left(P\right)+\underset{n\to \infty }{lim sup}\frac{1}{n}H\left(O\;P\left(n\right)|P^{\left(n\right)}\right)\\ \; & \leq \mathrm{KS}+\underset{n\to \infty }{lim sup}\frac{1}{n}H\left(O\;P\left(n\right)|P^{\left(n\right)}\right)\\ \; & \leq \mathrm{KS}+\underset{n\to \infty }{lim sup}\frac{1}{n}\sum_{\left(i,j\right)\in {\left(I\times J\right)}^{n}}\mu \left(P\left(\left(i,j\right)\right)\right)\log\left(\#\Delta \left(O\;P\left(n\right)|P\left(\left(i,j\right)\right)\right)\right), \end{array}$$

where we consider (i,j) itself as one multiindex and I×J as one index set. Thus,

$$P\left(\left(i,j\right)\right)=\overset{n-1}{\underset{t=0}{⋂}}T^{-t}\left(M_{i_{t}}\cap Q_{j_{t}}\right)$$

for all $\left(i,j\right)=\left(\left(i_{0},j_{0}\right),\left(i_{1},j_{1}\right),\ldots ,\left(i_{n-1},j_{n-1}\right)\right)\in {\left(I\times J\right)}^{n}$. Lemma A4 provides

(A10) $$\begin{array}{cc} \; & \sum_{\left(i,j\right)\in {\left(I\times J\right)}^{n}}\mu \left(P\left(\left(i,j\right)\right)\right)\cdot \log\left(\#\Delta \left(O\;P\left(n\right)|P\left(\left(i,j\right)\right)\right)\right)\\ \leq \log2 & \sum_{\left(i,j\right)\in {\left(I\times J\right)}^{n}}\mu \left(P\left(\left(i,j\right)\right)\right)\left(\sum_{u=1}^{m}\#\left\{s\in \left\{0,1,\ldots ,n-1\right\}|\left(i_{s},j_{s}\right)=\left(i_{n-u},j_{n-u}\right)\;\mathrm{and}\;s\neq n-u\right\}\right)\\ \leq \log2 & \sum_{\left(i,j\right)\in {\left(I\times J\right)}^{n}}\mu \left(P\left(\left(i,j\right)\right)\right)\left(\sum_{u=1}^{m}\#\left\{s\in \left\{0,1,\ldots ,n-1\right\}|j_{s}=j_{n-u}\;\mathrm{and}\;s\neq n-u\right\}\right)\\ =\log2 & \sum_{u=1}^{m}\sum_{j\in J^{n}}\left(\sum_{i\in I^{n}}\mu \left(P\left(\left(i,j\right)\right)\right)\right)\cdot \#\left\{s\in \left\{0,1,\ldots ,n-1\right\}|j_{s}=j_{n-u}\;\mathrm{and}\;s\neq n-u\right\}\\ =\log2 & \sum_{u=1}^{m}\sum_{j\in J^{n}}\mu \left(Q\left(j\right)\right)\cdot \#\left\{s\in \left\{0,1,\ldots ,n-1\right\}|j_{s}=j_{n-u}\;\mathrm{and}\;s\neq n-u\right\}\\ \leq \log2 & \sum_{u=1}^{m}\sum_{j\in J^{n}}\mu \left(Q\left(j\right)\right)\cdot \left(\#\left\{s\in \left\{0,1,\ldots ,n-u-1\right\}|j_{s}=j_{n-u}\right\}+u-1\right)\\ =\log2 & \cdot \left(m\left(m-1\right)/2+\sum_{u=1}^{m}\sum_{j\in J^{n}}\mu \left(Q\left(j\right)\right)\cdot \#\left\{s\in \left\{0,1,\ldots ,n-u-1\right\}|j_{s}=j_{n-u}\right\}\right). \end{array}$$

Combining (A9) and (A10) yields

$$\begin{array}{cc} \bar{\mathrm{PE}} & \leq \mathrm{KS}+\log2\cdot \sum_{u=1}^{m}\underset{n\to \infty }{lim sup}\frac{1}{n}\sum_{j\in J^{n}}\mu \left(Q\left(j\right)\right)\cdot \#\left\{s\in \left\{0,1,\ldots ,n-u-1\right\}|j_{s}=j_{n-u}\right\}. \end{array}$$

For each u∈{1,…,m}, we have

$$\begin{array}{cc} \; & \underset{n\to \infty }{lim sup}\frac{1}{n}\sum_{j\in J^{n}}\mu \left(Q\left(j\right)\right)\cdot \#\left\{s\in \left\{0,1,\ldots ,n-u-1\right\}|j_{s}=j_{n-u}\right\}\\ = & \underset{n\to \infty }{lim sup}\frac{1}{n}\sum_{j_{n-u}\in J}\sum_{j\in J^{n-u}}\mu \left(Q\left(\left(j,j_{n-u}\right)\right)\right)\cdot \#\left\{s\in \left\{0,1,\ldots ,n-u-1\right\}|j_{s}=j_{n-u}\right\}\\ = & \underset{n\to \infty }{lim sup}\frac{1}{n-u}\sum_{j_{n-u}\in J}\sum_{l=0}^{n-u}\mu \left(\left\{\omega \in T^{-n+u}\left(Q_{j_{n-u}}\right)|\#\left\{s\in \left\{0,1,\ldots ,n-u-1\right\}|T^{s}\left(\omega \right)\in Q_{j}\right\}=l\right\}\right)\cdot l\\ = & \sum_{Q\in Q}\underset{n\to \infty }{lim sup}\frac{1}{n-u}\sum_{l=0}^{n-u}\mu \left(T^{-n+u}\left(Q\right)\cap T^{-l}\left(Q\right)\right)\\ = & \sum_{Q\in Q}\underset{n\to \infty }{lim sup}\frac{1}{n-u}\sum_{l=0}^{n-u}\mu \left(Q\cap T^{-l}\left(Q\right)\right)\overset{\left(6\right)}{<}\epsilon . \end{array}$$

Hence,

$$\bar{\mathrm{PE}}\leq \mathrm{KS}+\log2\cdot m\cdot \epsilon .$$

The statement of the theorem follows from the fact that ϵ can be chosen arbitrarily close to 0.

**Lemma** **A5.** *Let* Ω *be a subset of R and A=B be the Borel σ-algebra on* Ω*. If T is (countably) piecewise monotone and completely periodic, i.e.,*

$$\mu \left(\overset{\infty }{\underset{t=1}{⋃}}\left\{\omega \in \Omega |T^{t}\left(\omega \right)=\omega \right\}\right)=1,$$

*then*

$$\bar{\mathrm{PE}}=0$$

*holds true.*

**Proof.** Let

$$\Theta_{k}:=\overset{k}{\underset{t=1}{⋃}}\left\{\omega \in \Omega :T^{t}\left(\omega \right)=\omega \right\}$$

be the set of all points with period smaller or equal to k∈N and

$$\Theta =\overset{\infty }{\underset{t=1}{⋃}}\left\{\omega \in \Omega :T^{t}\left(\omega \right)=\omega \right\}$$

the set of all periodic points. Since μ(Θ)=1, for all ϵ>0 there exists k∈N with

$$\mu \left(\Theta_{k}\right)>1-\epsilon .$$

This implies

$$\begin{array}{cc} \bar{\mathrm{PE}}\leq & \;\underset{n\to \infty }{lim sup}\frac{1}{n}H\left(O\;P\left(n\right)\vee \left\{\Theta_{k},\Omega \backslash \Theta_{k}\right\}\right)\\ = & \;\underset{n\to \infty }{lim sup}\frac{1}{n}\left[H\left(O\;P\left(n\right)\vee \left\{\Theta_{k},\Omega \backslash \Theta_{k}\right\}\right)-H\left(\Theta_{k},\Omega \backslash \Theta_{k}\right)\right] \end{array}$$

$$\begin{array}{cc} = & \;\mu \left(\Theta_{k}\right)\cdot \underset{n\to \infty }{lim sup}\frac{1}{n}-\sum_{P_{\pi }\in O\;P\left(n\right)}\frac{\mu (P_{\pi }\cap \Theta_{k})}{\mu (\Theta_{k})}\log\left(\frac{\mu (P_{\pi }\cap \Theta_{k})}{\mu (\Theta_{k})}\right)\\ \; & +\mu \left(\Omega \backslash \Theta_{k}\right)\cdot \underset{n\to \infty }{lim sup}\frac{1}{n}-\sum_{P_{\pi }\in O\;P\left(n\right)}\frac{\mu (P_{\pi }\backslash \Theta_{k})}{\mu (\Omega \backslash \Theta_{k})}\log\left(\frac{\mu (P_{\pi }\backslash \Theta_{k})}{\mu (\Omega \backslash \Theta_{k})}\right)\\ \overset{\left(A8\right)}{\leq } & \;\mu \left(\Theta_{k}\right)\cdot \underset{n\to \infty }{lim sup}\frac{1}{n}-\sum_{P_{\pi }\in O\;P\left(n\right)}\frac{\mu (P_{\pi }\cap \Theta_{k})}{\mu (\Theta_{k})}\log\left(\frac{\mu (P_{\pi }\cap \Theta_{k})}{\mu (\Theta_{k})}\right)\\ \; & +\epsilon \cdot \underset{n\to \infty }{lim sup}\frac{1}{n}\cdot \left[H\left(M^{\left(n\right)}\right)+\log\left(2\right)\cdot n\right]\\ \leq & \;\mu \left(\Theta_{k}\right)\cdot \underset{n\to \infty }{lim sup}\frac{1}{n}-\sum_{P_{\pi }\in O\;P\left(n\right)}\frac{\mu (P_{\pi }\cap \Theta_{k})}{\mu (\Theta_{k})}\log\left(\frac{\mu (P_{\pi }\cap \Theta_{k})}{\mu (\Theta_{k})}\right)+\left(H\left(M\right)+\log\left(2\right)\right)\cdot \epsilon \\ = & \;\mu \left(\Theta_{k}\right)\cdot \underset{n\to \infty }{lim sup}\frac{1}{n}-\sum_{P_{\pi }\in O\;P\left(k\right)}\frac{\mu (P_{\pi }\cap \Theta_{k})}{\mu (\Theta_{k})}\log\left(\frac{\mu (P_{\pi }\cap \Theta_{k})}{\mu (\Theta_{k})}\right)+\left(H\left(M\right)+\log\left(2\right)\right)\cdot \epsilon \\ = & \;(H(M)+\log(2))\cdot \epsilon . \end{array}$$

This provides $\bar{\mathrm{PE}}=0$ because ϵ can be chosen arbitrarily close to 0. □

**Remark** **A1.**
*To be able to show that Condition 2 holds true under the assumptions of Corollary 1 for non-ergodic systems via ergodic decomposition, one needs to require that (Ω,B,μ) is a Lebesgue space. A probability space (Ω,B,μ) is called a Lebesgue space if (Ω,B,μ) is isomorph to some probability space $\left(\tilde{\Omega },\tilde{B},\tilde{\mu }\right)$, where $\tilde{\Omega }$ is a complete separable metric space and $\tilde{B}$ the completion of the Borel σ-algebra on $\tilde{\Omega }$, i.e., $\tilde{B}$ contains additionally all subsets of Borel sets with measure 0. If Ω⊆R is a Borel subset, (Ω,B,μ) is a Lebesgue space if B is complete with regard to μ (see e.g., [14]).*
*Alternatively, one can use Rokhlin–Halmos towers to show that Condition 2 holds true for non-ergodic systems (see [10]). For this approach, it is only necessary to require that* Ω *is a separable metric space, B the Borel σ-algebra on Ω, and T:Ω→Ω an aperiodic map [15].*
*Moreover, notice that, in [10], it was required that* Ω *is a compact metric space so that μ is regular, which allowed for approximating any set of B by a finite union of intervals. However, this is not necessary because the Borel σ-algebra is generated by the algebra containing all sets of the type I∩Ω, where I is an open or closed interval, and every set of a σ-algebra can be approximated by a set of the algebra that generates that σ-algebra (see, e.g., [16]).*

### Appendix A.5. Proof of Lemma 1 and the ‘Ergodic Part’ of Lemma 2

Let Ω be a subset of R and A=B be the Borel σ-algebra on Ω. We start with showing Lemma 1. For this, fix some n∈N. By its definition, the set $V_{n}$ can be written as a union of sets in $O\;P\left(n\right)\vee T^{-1}\left(O\;P\left(n\right)\right)$. Notice that

(A11) $$O\;P\left(n+1\right)=O\;P\left(n\right)\vee T^{-1}\left(O\;P\left(n\right)\right)\vee {\left(id,T^{n}\right)}^{-1}\left(\left\{R,\Omega^{2}\backslash R\right\}\right).$$

For $Q\in O\;P\left(n\right)\vee T^{-1}\left(O\;P\left(n\right)\right)$, consider some ω∈Q. If $\omega \notin V_{n}$ is true, we can use the transitivity of the order relation to determine the order relation of ω and $T^{n}\left(\omega \right)$ from the ordering given by *Q*. This implies

(A12) $$\#\Delta ({\left(id,T^{n}\right)}^{-1}\left(\left\{R,\Omega^{2}\backslash R\right\}\right)|Q)=1$$

for all $Q\subseteq \Omega \backslash V_{n}$. Thus,

$$\begin{array}{cc} & H(O\;P\left(n+1\right)|O\;P\left(n\right)\vee T^{-1}\left(O\;P\left(n\right)\right))\\ \overset{\left(A11\right)}{\leq } & \;H(O\;P\left(n\right)\vee T^{-1}\left(O\;P\left(n\right)\right)|O\;P\left(n\right)\vee T^{-1}\left(O\;P\left(n\right)\right))+\\ & \;H({\left(id,T^{n}\right)}^{-1}\left(\left\{R,\Omega^{2}\backslash R\right\}\right)|O\;P\left(n\right)\vee T^{-1}\left(O\;P\left(n\right)\right))\\ = & \;H({\left(id,T^{n}\right)}^{-1}\left(\left\{R,\Omega^{2}\backslash R\right\}\right)|O\;P\left(n\right)\vee T^{-1}\left(O\;P\left(n\right)\right))\\ \overset{\left(A1\right)}{\leq } & \;\sum_{Q\in O\;P\left(n\right)\vee T^{-1}\left(O\;P\left(n\right)\right)}\mu \left(Q\right)\cdot \log\left(\#\Delta \left({\left(id,T^{n}\right)}^{-1}\left(\left\{R,\Omega^{2}\backslash R\right\}\right)|Q\right)\right)\\ = & \;\sum_{\begin{array}{c} Q\in O\;P\left(n\right)\vee T^{-1}\left(O\;P\left(n\right)\right)\\ Q\subseteq V_{n} \end{array}}\mu \left(Q\right)\cdot \log\left(\#\Delta \left({\left(id,T^{n}\right)}^{-1}\left(\left\{R,\Omega^{2}\backslash R\right\}\right)|Q\right)\right)\\ & +\sum_{\begin{array}{c} Q\in O\;P\left(n\right)\vee T^{-1}\left(O\;P\left(n\right)\right)\\ Q⊈V_{n} \end{array}}\mu \left(Q\right)\cdot \log\left(\#\Delta \left({\left(id,T^{n}\right)}^{-1}\left(\left\{R,\Omega^{2}\backslash R\right\}\right)|Q\right)\right)\\ = & \;\sum_{\begin{array}{c} Q\in O\;P\left(n\right)\vee T^{-1}\left(O\;P\left(n\right)\right)\\ Q\subseteq V_{n} \end{array}}\mu \left(Q\right)\cdot \log\left(\#\Delta \left({\left(id,T^{n}\right)}^{-1}\left(\left\{R,\Omega^{2}\backslash R\right\}\right)|Q\right)\right)\\ & +\sum_{\begin{array}{c} Q\in O\;P\left(n\right)\vee T^{-1}\left(O\;P\left(n\right)\right)\\ Q\subseteq \Omega \backslash V_{n} \end{array}}\mu \left(Q\right)\cdot \log\left(\#\Delta \left({\left(id,T^{n}\right)}^{-1}\left(\left\{R,\Omega^{2}\backslash R\right\}\right)|Q\right)\right)\\ \overset{\left(A12\right)}{=} & \;\sum_{\begin{array}{c} Q\in O\;P\left(n\right)\vee T^{-1}\left(O\;P\left(n\right)\right)\\ Q\subseteq V_{n} \end{array}}\mu \left(Q\right)\cdot \log\left(\#\Delta \left({\left(id,T^{n}\right)}^{-1}\left(\left\{R,\Omega^{2}\backslash R\right\}\right)|Q\right)\right)\\ \leq & \;\sum_{\begin{array}{c} Q\in O\;P\left(n\right)\vee T^{-1}\left(O\;P\left(n\right)\right)\\ Q\subseteq V_{n} \end{array}}\mu \left(Q\right)\cdot \log\left(2\right)\\ = & \;\log\left(2\right)\cdot \mu \left(V_{n}\right). \end{array}$$

This shows Lemma 1.

To prove the ‘ergodic part’ of Lemma 2, take ϵ>0. Choose an ordered partition $U={\left\{U_{i}\right\}}_{i=1}^{N}$ of Ω such that $0<\mu (U_{i})<\epsilon$ holds true for all i∈I. This is always possible because μ was assumed to be aperiodic. Label the sets $U_{i}\in U$ with i∈{1,2,…,N} in such a way that

$$i_{1}<i_{2}\Rightarrow \mu^{2}\left(\left\{\left(\omega_{1},\omega_{2}\right)\in U_{i_{1}}\times U_{i_{2}}:\omega_{1}>\omega_{2}\right\}\right)=0$$

holds true for all $i_{1},i_{2}\in \left\{1,2,\ldots ,N\right\}$. Since *T* is ergodic, there exists an $n_{o}\in N$ such that

$$\mu \left(\overset{N}{\underset{i=1}{⋂}}\overset{n_{0}-1}{\underset{s=1}{⋃}}T^{-s}\left(U_{i}\right)\right)>1-\epsilon$$

holds true.

The set ${⋂}_{i=1}^{N}{⋃}_{s=1}^{n_{0}-1}T^{-s}\left(U_{i}\right)$ consists of all ω∈Ω with orbit $\left(\omega ,T\left(\omega \right),\ldots ,T^{n_{0}-1}\right)$ visiting each of the sets in U. Thus, if such ω lies in $U_{i}$ with 1<i<N and in $V_{t}$ for $t\geq n_{0}$, by definition of $V_{t}$, the point $T^{t}\left(\omega \right)$ must belong to $U_{t-1}\cup U_{t}\cup U_{t+1}$. With a similar argumentation for $\omega \in U_{1}$ or $\omega \in U_{N}$, one obtains the following:

$$\begin{array}{cc} \; & \mu \left(V_{t}\cap \overset{N}{\underset{i=1}{⋂}}\overset{n_{0}-1}{\underset{s=1}{⋃}}T^{-s}\left(U_{i}\right)\right)\\ \; & \leq \mu \left(U_{1}\cap T^{-t}\left(U_{1}\cup U_{2}\right)\right)+\sum_{i=2}^{N-1}\mu \left(U_{i}\cap T^{-t}\left(U_{i-1}\cup U_{i}\cup U_{i+1}\right)\right)+\mu \left(U_{N}\cap T^{-t}\left(U_{N-1}\cup U_{N}\right)\right) \end{array}$$

holds true for all t∈N with $t>n_{0}$. Using the ergodicity of *T* implies

$$\begin{array}{cc} \; & \lim_{n\to \infty }\frac{1}{n}\sum_{t=1}^{n}\mu \left(V_{t}\cap \overset{N}{\underset{i=1}{⋂}}\overset{n_{0}-1}{\underset{s=1}{⋃}}T^{-s}\left(U_{i}\right)\right)=\lim_{n\to \infty }\frac{1}{n}\sum_{t=1}^{n}\mu \left(V_{t+n_{0}}\cap \overset{N}{\underset{i=1}{⋂}}\overset{n_{0}-1}{\underset{s=1}{⋃}}T^{-s}\left(U_{i}\right)\right)\\ \; & \leq \mu \left(U_{1}\right)\mu \left(U_{1}\cup U_{2}\right)+\sum_{i=2}^{N-1}\mu \left(U_{i}\right)\mu \left(U_{i-1}\cup U_{i}\cup U_{i+1}\right)+\mu \left(U_{N}\right)\mu \left(U_{N-1}\cup U_{N}\right)\\ \; & \leq \mu \left(U_{1}\right)2\epsilon +\sum_{i=2}^{N-1}\mu \left(U_{i}\right)3\epsilon +\mu \left(U_{N}\right)2\epsilon \\ \; & \leq 3\epsilon . \end{array}$$

The Stolz–Cesàro theorem then provides

$$\begin{array}{cc} \; & \underset{n\to \infty }{lim inf}\mu \left(V_{n}\cap \overset{N}{\underset{i=1}{⋂}}\overset{n_{0}-1}{\underset{s=1}{⋃}}T^{-s}\left(U_{i}\right)\right)\leq \underset{n\to \infty }{lim inf}\frac{1}{n}\sum_{t=1}^{n}\mu \left(V_{t}\cap \overset{N}{\underset{i=1}{⋂}}\overset{n_{0}-1}{\underset{s=1}{⋃}}T^{-s}\left(U_{i}\right)\right)\leq 3\epsilon . \end{array}$$

Hence,

$$\begin{array}{cc} \; & \underset{n\to \infty }{lim inf}\mu \left(V_{n}\right)\\ \; & =\underset{n\to \infty }{lim inf}\left[\mu \left(V_{n}\cap \overset{N}{\underset{i=1}{⋂}}\overset{n_{0}-1}{\underset{s=1}{⋃}}T^{-s}\left(U_{i}\right)\right)+\mu \left(V_{n}\backslash \overset{N}{\underset{i=1}{⋂}}\overset{n_{0}-1}{\underset{s=1}{⋃}}T^{-s}\left(U_{i}\right)\right)\right]\\ \; & \leq \underset{n\to \infty }{lim inf}\mu \left(V_{n}\cap \overset{N}{\underset{i=1}{⋂}}\overset{n_{0}-1}{\underset{s=1}{⋃}}T^{-s}\left(U_{i}\right)\right)+\epsilon \\ \; & \leq 4\epsilon . \end{array}$$

Since ϵ can be choosen arbitrarily close to 0, this implies ${lim inf}_{n\to \infty }\mu \left(V_{n}\right)=0$.

### Appendix A.6. Proof of Theorem 5

Let Ω be a subset of R and A=B be the Borel σ-algebra on Ω and consider the set

$$\Theta =\overset{\infty }{\underset{t=1}{⋃}}\left\{\omega \in \Omega |T^{t}\left(\omega \right)=\omega \right\}.$$

Since *T* is μ-almost surely aperiodic, we have μ(Θ)=0.

We will prove the statement of the theorem by contradiction. Suppose $\sum_{n=1}^{\infty }\mu \left(V_{n}\right)<\infty$ holds true. Using the Borel–Cantelli lemma, this implies

$$\mu \left(\overset{\infty }{\underset{k=1}{⋂}}\overset{\infty }{\underset{n=k}{⋃}}V_{n}\right)=0$$

or, equivalently,

$$\lim_{K\to \infty }\mu \left(\overset{K}{\underset{k=1}{⋃}}\overset{\infty }{\underset{n=k}{⋂}}\left(\Omega \backslash V_{n}\right)\right)=\mu \left(\overset{\infty }{\underset{k=1}{⋃}}\overset{\infty }{\underset{n=k}{⋂}}\left(\Omega \backslash V_{n}\right)\right)=1.$$

Therefore, there exists a K∈N with

$$\mu \left(\left(\overset{\infty }{\underset{n=K}{⋂}}\left(\Omega \backslash V_{n}\right)\right)\backslash \Theta \right)=\mu \left(\overset{\infty }{\underset{n=K}{⋂}}\left(\Omega \backslash V_{n}\right)\right)=\mu \left(\overset{K}{\underset{k=1}{⋃}}\overset{\infty }{\underset{n=k}{⋂}}\left(\Omega \backslash V_{n}\right)\right)>0.$$

Set

$$\delta \left(\omega \right):=\min_{1\leq s\leq K-1}\left|\omega -T^{s}\left(\omega \right)\right|$$

for all ω∈Ω. Notice that every aperiodic point ω∉Θ satisfies δ(ω)>0. Thus,

$$\begin{array}{cc} 0 & <\mu \left(\overset{\infty }{\underset{n=K}{⋂}}\left(\Omega \backslash V_{n}\right)\backslash \Theta \right)=\mu \left(\overset{\infty }{\underset{i=1}{⋃}}\left\{\omega \in \overset{\infty }{\underset{n=K}{⋂}}\left(\Omega \backslash V_{n}\right)\backslash \Theta |\delta \left(\omega \right)>1/i\right\}\right)\\ \; & =\lim_{i\to \infty }\mu \left(\left\{\omega \in \overset{\infty }{\underset{n=K}{⋂}}\left(\Omega \backslash V_{n}\right)\backslash \Theta |\delta \left(\omega \right)>1/i\right\}\right). \end{array}$$

Thus, there exists some δ>0 such that

$$A_{\delta }:=\left\{\omega \in \overset{\infty }{\underset{n=K}{⋂}}\left(\Omega \backslash V_{n}\right)\backslash \Theta |\delta \left(\omega \right)>\delta \right\}$$

has a strictly positive measure. Because there exists a countable set $\Omega_{\delta }\subseteq \Omega$ with

$$A_{\delta }=\underset{\omega \in \Omega_{\delta }}{⋃}A_{\delta }\cap \left(\omega -\delta /2,\omega +\delta /2\right),$$

we have

$$\mu (A_{\delta }\cap \left(\omega_{0}-\delta /2,\omega_{0}+\delta /2\right))>0$$

for some $\omega_{0}\in \Omega$. Using the ergodicity of *T*, this implies

$$\begin{array}{c} \mu \left(\overset{\infty }{\underset{n=K}{⋃}}T^{-n}\left(\left(\omega_{0}-\delta /2,\omega_{0}+\delta /2\right)\right)\right)=\mu \left(\overset{\infty }{\underset{n=0}{⋃}}T^{-n}\left(T^{-K}\left(\left(\omega_{0}-\delta /2,\omega_{0}+\delta /2\right)\right)\right)\right)=1 \end{array}$$

and, consequently,

$$\begin{array}{cc} \; & \mu \left(A_{\delta }\cap \left(\omega_{0}-\delta /2,\omega_{0}+\delta /2\right)\cap \overset{\infty }{\underset{n=K}{⋃}}T^{-n}\left(\left(\omega_{0}-\delta /2,\omega_{0}+\delta /2\right)\right)\right)\\ \; & =\mu (A_{\delta }\cap \left(\omega_{0}-\delta /2,\omega_{0}+\delta /2\right))>0. \end{array}$$

Thus, in particular, $A_{\delta }\cap \left(\omega_{0}-\delta /2,\omega_{0}+\delta /2\right)\cap {⋃}_{n=K}^{\infty }T^{-n}\left(\left(\omega_{0}-\delta /2,\omega_{0}+\delta /2\right)\right)$ is not empty. Now take some

$$\omega \in A_{\delta }\cap \left(\omega_{0}-\delta /2,\omega_{0}+\delta /2\right)\cap \overset{\infty }{\underset{n=K}{⋃}}T^{-n}\left(\left(\omega_{0}-\delta /2,\omega_{0}+\delta /2\right)\right).$$

We have $|\omega -\omega_{0}|<\delta /2$. Additionally, there exists $n_{0}\in N$ with $n_{0}\geq K$ such that $T^{n_{0}}\left(\omega \right)\in \left(\omega_{0}-\delta /2,\omega_{0}+\delta /2\right)$ holds true, which is equivalent to $|\omega_{0}-T^{n_{0}}\left(\omega \right)|<\delta /2$. As a consequence,

$$|\omega -T^{n_{0}}\left(\omega \right)\left|\leq \right|\omega -\omega_{0}\left|+\right|\omega_{0}-T^{n_{0}}\left(\omega \right)|<\delta$$

holds true. This implies that

$$m:=\min\{n\in N||\omega -T^{n}\left(\omega \right)|<\delta \}$$

is smaller or equal to $n_{0}$. In particular, m∈N is well defined and not infinite. On the other hand,

$$\omega \in A_{\delta }\subseteq \{\omega \in \Omega |\min_{1\leq s\leq K-1}|\omega -T^{s}\left(\omega \right)\left|>\delta \right\}$$

implies m≥K. By construction of *m*, we have

$$|\omega -T^{s}\left(\omega \right)\left|\geq \delta >\right|\omega -T^{m}\left(\omega \right)|$$

for all s∈{1,2,…,m−1}. Hence, $\omega \in V_{m}$ holds true, which is a contradiction to

$$\omega \in A_{\delta }\subseteq \overset{\infty }{\underset{n=K}{⋂}}\Omega \backslash V_{n}.$$

Therefore, $\sum_{n=1}^{\infty }\mu \left(V_{n}\right)<\infty$ cannot be true.
